# A protocol for remote collection of skeletal muscle mass via D_3_-creatine dilution in community-dwelling postmenopausal women from the Women’s Health Initiative

**DOI:** 10.1371/journal.pone.0300140

**Published:** 2024-04-17

**Authors:** Hailey R. Banack, Jean Wactawski-Wende, Heather M. Ochs-Balcom, Elizabeth M. Cespedes Feliciano, Bette Caan, Catherine Lee, Garnet Anderson, Mahalakshmi Shankaran, William J. Evans

**Affiliations:** 1 Epidemiology Division, Dalla Lana School of Public Health, University of Toronto, Toronto, Ontario, Canada; 2 Department of Epidemiology and Environmental Health, School of Public Health and Health Professions, State University of New York at Buffalo, Buffalo, NY, United States of America; 3 Department of Health Systems Science, Kaiser Permanente Bernard J. Tyson School of Medicine, Pasadena, CA, United States of America; 4 Fred Hutchinson Cancer Research Center; 5 Department of Nutritional Sciences and Toxicology, University of California, Berkeley, CA, United States of America; 6 Division of Geriatrics, Duke University Medical Center, Durham, NC, United States of America; National Institute of Biomedical Innovation Health and Nutrition, JAPAN

## Abstract

**Background:**

There is emerging evidence that cancer and its treatments may accelerate the normal aging process, increasing the magnitude and rate of decline in functional capacity. This accelerated aging process is hypothesized to hasten the occurrence of common adverse age-related outcomes in cancer survivors, including loss of muscle mass and decrease in physical function. However, there is no data describing age-related loss of muscle mass and its relation to physical function in the long-term in cancer survivors.

**Methods:**

This study protocol describes the use of a novel method of muscle mass measurement, D3-creatine dilution method (D_3_Cr), in a large sample (n~6000) of community dwelling postmenopausal women from the Women’s Health Initiative (WHI). D_3_Cr will be used to obtain a direct measure of muscle mass remotely. Participants will be drawn from two sub-cohorts embedded within the WHI that have recently completed an in-home visit. Cancer survivors will be drawn from the Life and Longevity After Cancer (LILAC) cohort, and cancer-free controls will be drawn from the WHI Long Life Study 2. The overall objective of this study is to examine the antecedents and consequences of low muscle mass in cancer survivors. The study aims are to: 1) create age-standardized muscle mass percentile curves and z-scores to characterize the distribution of D3- muscle mass in cancer survivors and non-cancer controls, 2) compare muscle mass, physical function, and functional decline in cancer survivors and non- cancer controls, and 3) use machine learning approaches to generate multivariate risk-prediction algorithms to detect low muscle mass.

**Discussion:**

The D_3_Cr method will transform our ability to measure muscle mass in large-scale epidemiologic research. This study is an opportunity to advance our understanding of a key source of morbidity among older and long-term female cancer survivors. This project will fill knowledge gaps, including the antecedents and consequences of low muscle mass, and use innovative methods to overcome common sources of bias in cancer research. The results of this study will be used to develop interventions to mitigate the harmful effects of low muscle mass in older adults and promote healthy survivorship in cancer survivors in the old (>65) and oldest-old (>85) age groups.

## Background

Oncology researchers have identified muscle mass as a priority biomarker for cancer research [1]. There is compelling evidence that muscle quantity and function increases morbidity and mortality after a diagnosis of cancer, including both cancer-specific and overall mortality, risk of non-cancer comorbid conditions, and functional decline [2–7]. Low muscle size (area) is highly prevalent in patients diagnosed with cancer: prior studies of nonmetastatic breast and colorectal cancer patients have found 30% and 42% breast and colorectal cancer patients have low muscle size at diagnosis [8]. Muscle likely influences cancer-related outcomes through multiple physiologic, metabolic and functional pathways [9]. Proposed mechanisms include the important contribution of skeletal muscle in metabolism and systemic inflammation: skeletal muscle is the largest organ in the body and secretes cytokines and other peptides (known as myokines) that have autocrine, paracrine, or endocrine actions [10]. Muscle size also influences the pharmacokinetics of chemotherapy and is associated with dose-limiting toxicity [11], thereby reducing the effectiveness of these life-saving cancer therapies among individuals with low muscle mass.

After diagnosis, several factors increase the risk of muscle loss among cancer survivors, including direct effects of treatment on protein synthesis and degradation, alterations in nutrient intake due to nausea, and changes in energy expenditure due to inactivity or bedrest [12]. Despite growing knowledge of the importance of muscle mass at diagnosis in cancer patients, little is known about longer-term changes in muscle mass in cancer survivors. This manuscript describes a study protocol for a project that will measure muscle mass in female cancer survivors. In this project, we will use an innovate approach to measure muscle mass remotely, via the D3-Creatine (D_3_Cr) method. The overall aim of this project is to add to our understand of the antecedents and consequences of muscle mass in cancer survivors.

Research is urgently needed to study older cancer survivors as they represent a growing segment of the population. Given increasing rates of cancer survivorship, and increasing life expectancies (in women in particular), the number of older female cancer survivors is rising [13]. Research focused on muscle mass in older women is particularly relevant because, on average, postmenopausal women lose 1–3% of muscle mass and strength per year [14]. Although muscle mass is recognized as an important predictor of morbidity and mortality in older adults, measurement of muscle mass in large-scale population health and epidemiologic research remains a challenge [15–17]. Imaging modalities (e.g., DXA, CT, MRI) exist for measurement of muscle mass in clinical settings and can be used for diagnostic purposes or clinical research studies [18]. However, there are numerous barriers to implementation of these measurement approaches in population health research, including cost, accessibility, staffing needs, and for CT scans, radiation exposure.

Recently, the creatine dilution (D_3_Cr) method has gained popularity as a measure of muscle mass in large-scale, population-based research studies [19, 20]. The D_3_Cr method is a simple, inexpensive, and non-invasive assessment that can be used to measure muscle mass remotely in a community dwelling population of postmenopausal women. It has been validated through rigorous laboratory and clinical testing and previously used in clinic-based epidemiology research [19, 21, 22]. Cross-sectional and longitudinal validation studies have been performed in rodents [23] and humans [24]. Dilution of a stable isotope-labeled creatine, and measure of urinary creatinine enrichment provides a direct measure of whole body creatine pool size which is proportional to total body muscle mass [21].

Many women experience changes loss of muscle mass as part of the normal aging process [25]. However, there are several key unanswered questions about whether postmenopausal cancer survivors experience greater declines in muscle mass compared to women without a history of cancer. If yes, do they undergo an accelerated aging trajectory, with a different slope and quicker progression to functional impairment and disability? This protocol will describe the design of a prospective longitudinal study using the D_3_Cr method to measure muscle mass in postmenopausal cancer survivors and a comparison group of women without a cancer history (“non-cancer controls”). Both cancer survivors and cancer-free controls are drawn from the same source population, the Women’s Health Initiative (WHI).The specific objectives of this project are to: 1) develop age-standardized percentile curves to characterize the distributions of D_3_- muscle mass in cancer survivors and cancer-free controls, 2) examine whether the associations between muscle mass, physical function, and functional decline differ in cancer survivors and cancer-free controls, and 3) create multivariate risk-prediction algorithms to detect low muscle mass in cancer survivors and cancer-free controls. We hypothesize that cancer survivors will have lower muscle mass and experience accelerated decline in physical function compared to cancer-free controls.

## Materials and methods

### Study population

The Ms. LILAC study (**M**u**s**cle mass in the **Li**fe and **L**ongevity **A**fter **C**ancer cohort) will include approximately 6000 postmenopausal women. Participants will be recruited from the ongoing Women’s Health Initiative (WHI) study cohort. The WHI is a prospective longitudinal study focused on examining causes of morbidity and mortality in postmenopausal women.

All Ms. LILAC participants have been active participants in the WHI study since the 1990s. Postmenopausal women aged 50–79 years enrolled in the WHI clinical trials and observational study between 1993 and 1998 at 40 clinical centers across the U.S. (n = 161,808) [26]. Over the past thirty years, follow-up has been consistent and is still ongoing. In the initial phase of the WHI (up to 2005), participants visited clinical centers for in-person measurements, including anthropometric measurements. Since then, the WHI has collected comprehensive annual questionnaires on medical history, demographics, and lifestyle characteristics via mail, online surveys and telephone [27].

**Fig 1:**
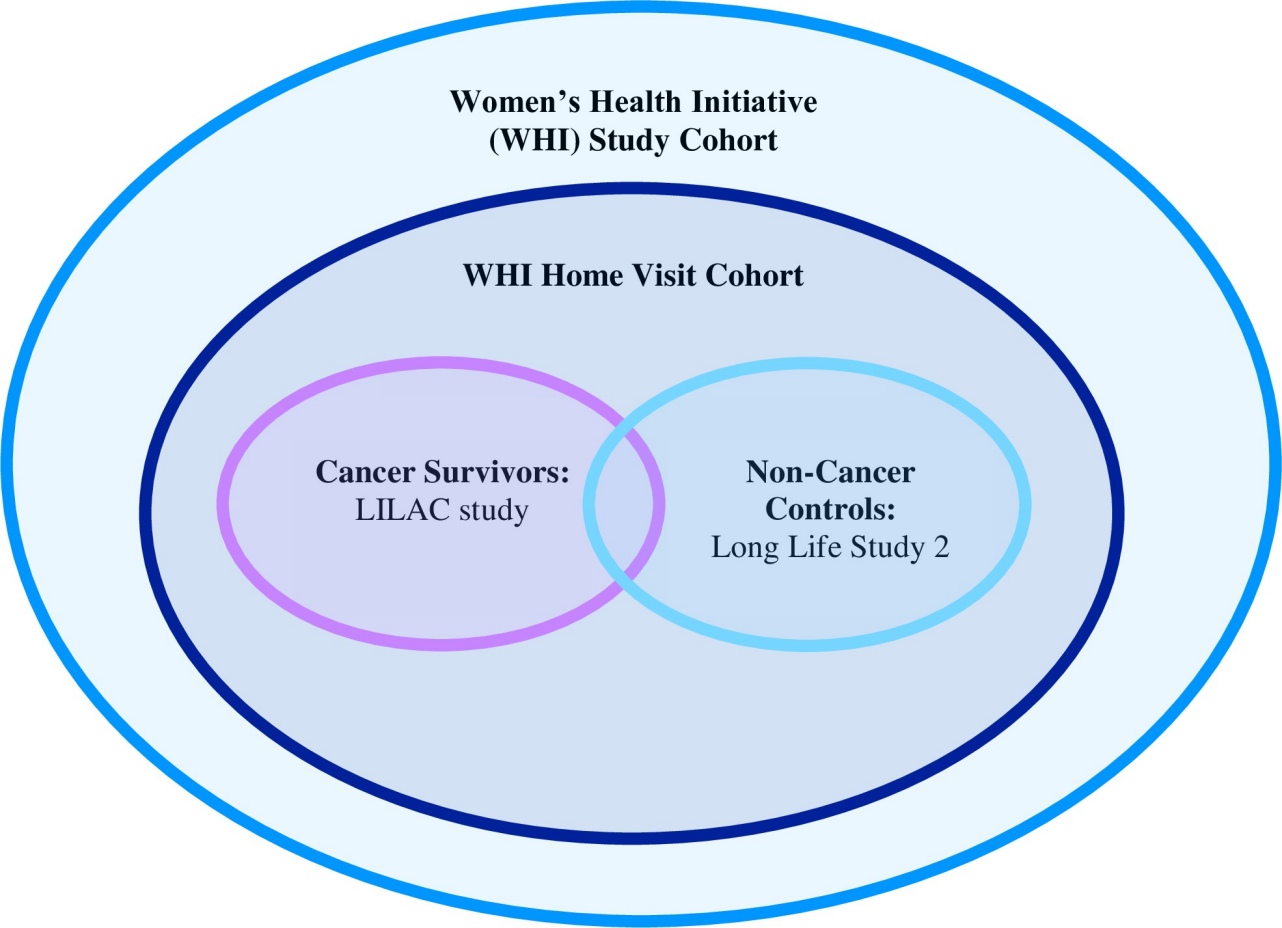
Description of the study population for Ms. LILAC comprised of WHI sub-cohorts Long Life Study 2 (LLS2) and LILAC (Life and Longevity After Cancer cohort).

For Ms. LILAC, we will recruit women who are actively still participating in the WHI and have recently completed an in-home study funded by the WHI. The sample size for Ms. LILAC is limited to all women who have completed the WHI home visit, as the home visit will provide important information on measured covariates (i.e., body weight, blood-based biomarkers, measures of physical function). The study population is depicted in Fig 1. Cancer survivors will be recruited from a WHI sub-cohort called the Life and Longevity After Cancer (LILAC) study. Postmenopausal women with no cancer history (“non-cancer controls”) will be recruited from a WHI sub-cohort called the Long Life Study 2 (LLS2), a minority enriched sub-cohort of women [28, 29]. The WHI is currently completing in-home study visits for LILAC and LLS2 participants. Home visits consist of anthropometric measures (e.g., height, weight, blood pressure), assessment of physical function via the short physical performance battery (SPPB), and a fasting venous blood draw [30].

### LILAC (Life and Longevity After Cancer) cohort

LILAC is a cohort of cancer survivors embedded within the WHI. Recruitment protocols for LILAC include: WHI participants who initially self-report a cancer diagnosis and self-report is confirmed by review of medical records by trained physician adjudicators. All data is coded using NCI SEER (Surveillance, Epidemiology, and End Results) standards [28]. LILAC participants who are survivors of breast, lung, colorectal, or endometrial cancer are currently being recruited for home visits.

Information on cancer type, tumor characteristics (e.g., molecular testing, stage and grade), cancer treatments and outcomes are collected as part of LILAC. Medical records are reviewed by trained physician adjudicators to confirm the previously documented diagnostic information and records information on surgery type and date(s); tumor characteristics (HER2, estrogen receptor status); types and start dates of neoadjuvant and adjuvant chemotherapy; radiotherapy along with total dose; biologic therapies; and hormonal/endocrine therapies and enters these directly into the WHI extension study database. For WHI participants enrolled in fee-for-service Medicare at the time of their cancer diagnosis, WHI obtains treatment and recurrence through Medicare linkage. For participants with cancer who were not covered by fee-for-service Medicare at time of diagnosis, WHI collects medical records to obtain comparable data. In addition, LILAC surveys collect self-reported information on initial cancer treatment including surgery type, chemotherapy, hormonal therapy, radiation therapy, biological therapies and complementary/alternative medicine, including type, start dates, and providers of these treatments; new cancer events related to their primary cancer diagnosis; longer-term disabling conditions; effects on quality-of-life that are common sequelae to many cancers or their treatments but not covered by WHI questionnaires (e.g., lymphedema, cardiotoxicity, nephrotoxicity, neurotoxicity); and questions on insurance coverage, income, employment status, access to care, recurrence and health outcomes such as anxiety, depression, overall quality of life, and self-rating of health [31–34]. These rich data will allow us to fully characterize and control for heterogeneity by cancer site and severity, enabling meaningful analyses both overall and when stratified by cancer site or stage.

### LLS2 (Long Life Study 2)

Postmenopausal women with no history of cancer (“cancer-free controls”) will be recruited from the Long Life Study 2 (LLS2), a WHI sub-cohort. The LLS2 sub-cohort consists of women who completed an in-home visit in 2012–2013 as part of the initial Long Life Study [29], are still alive and participating in the WHI, and are capable of providing informed consent. All 7,875 women who had a home visit as part of the initial Long-Life Study (2012–2013) are invited by mail to participate in the Long-Life Study 2 (2022–2024). The mailed invitation packet consists of a letter explaining the nature and purpose of the study, written informed consent form, and a pre-paid return mailer. Participation in the LLS2 includes an in-home study visit by a trained examiner. The protocol for the home visits includes the same measures as completed as part of the first LLS home visit.

### Informed consent

All WHI participants provided written informed consent at initial study recruitment (1993–1998). The informed consent process for the WHI home visits is managed by the WHI Clinical Coordinating Center and approved by the IRB at the Fred Hutchinson Cancer Center. All participants must provide written informed consent prior to the in-home visit. As part of the informed consent process, the WHI Clinical Coordinating Center asks participants if they are willing to allow WHI to share their contact information with WHI ancillary studies, such as Ms. LILAC (“consent to contact” process). If a participant agrees, their contact information is shared with the Ms. LILAC team data manager via a secure data portal. The informed consent process for the Ms. LILAC study, including the consent to contact process, and the Ms. LILAC complete study protocol, was reviewed and approved by the Institutional Review Board at the University at Buffalo. Upon receipt of the participant’s name and contact information at the University at Buffalo, the Ms. LILAC team sends a mailed invitation letter to ask them to take part in our study; this invitation packet includes a written informed consent form for participants to sign and return to the study team. A copy of the University at Buffalo IRB-approved Ms. LILAC invitation letter is included in the supplementary materials (S1 File) accompanying this manuscript.

### Ms. LILAC study overview

All contact for the Ms. LILAC study will occur remotely, via telephone and mail, after the WHI home visit has been completed for both LILAC and LLS2 participants [28, 29]. Data collection for Ms. LILAC (D_3_Cr muscle mass) is currently underway and scheduled to be completed by 2025. We will measure D_3_Cr muscle mass at one time point shortly after completion of the WHI home visits. Annual WHI questionnaires will assess self-reported functional outcomes over time, including ability to complete activities of daily, disability, and mobility limitation, enabling examination of muscle mass as a predictor of these longitudinal outcomes.

### Protocol for remote measurement of D_3_-Creatine (D_3_Cr)

Study participants are sent a kit to measure muscle mass using the D_3_Cr method at home. The D_3_Cr capsule includes deuterated creatine (25mg) produced by Cambridge Isotope Laboratories (Cambridge, MA). Greenpark Compounding Pharmacy (Houston, TX) uses the D_3_Cr to create the study capsules. The boxed kit will include all supplies and instructions with pictures. Study kits will include a urine collection cup, a collection dipstick labeled with participant ID, a clear ziptop bag, freezer pack, and pre-addressed return mailer.

Typically, participants are instructed to take the D_3_Cr pill (capsule) within a week of receiving the study kit by mail. Participants are asked to collect a single fasting morning sample using a urine dipstick provided by the study team. Participants are instructed to take the capsule on Saturday morning and collect their urine sample on Tuesday morning. On Monday afternoon, project staff make telephone calls to participants to remind them to fast overnight in preparation for sample collection. According to laboratory validation studies, participants should collect the urine sample between 48–96 hours after ingesting the D_3_Cr capsule [24]. Once the sample is collected, it is stored in an insulated mailing package with ice packs to ensure the dipstick is kept cold. Participants are asked to call to schedule a courier pick-up before noon to schedule a same-day at-home pick-up. The study team provides pre-addressed mailing labels affixed to the outside of the insulated mailing package. Once the package is picked up, it is returned to the study team via overnight shipping, with guaranteed next morning delivery.

Once samples are delivered to the study team, a project staff member opens the package and documents the condition of each sample. Each participant is assigned a unique study ID code for tracking purposes; de-identified participant data on sample condition and measurements are stored in a password-protected Microsoft Access database. Dipsticks are labeled with a unique participant ID number and placed in -20°C freezers in the university (departmental) biorepository. Frozen samples will be sent in batches on dry ice using priority overnight shipping to the University of California, Berkeley for analysis. We tested this procedure during our pilot study and all samples were transported in good condition with no spoilage [22]. As part of the study protocol, there are several reminder forms and instruction sheets shared with study participants. These documents are available as supplementary materials (S1 File) linked to this article and may be used or modified as needed for other study designs and contexts.

### Laboratory measurement of D_3_-creatinine enrichment

D_3_-creatinine enrichment is measured by liquid chromatography with tandem mass spectrometry. The laboratory protocols and details of the sample processing have been previously described [17, 21, 23, 24]. Samples are run in triplicate and average values are reported. Samples with CVs greater than 5% will be subjected to reanalysis. The total body creatine pool size is calculated as retained (delivered) D_3_Cr dose divided by D_3_Crn enrichment and muscle mass [21]. A small, variable amount of the D_3_Cr label “spills” into urine during the first hours after ingestion of the D_3_Cr capsule in some individuals, an algorithm is used to correct for loss of label using the fasting urine Cr/Crn ratio [24].

### Data management

Study data will be stored on secure, password protected computers accessible only by Ms. LILAC study team members. Once the study is completed, data on D_3_Cr muscle mass collected in the Ms. LILAC study will be de-identified and linked to the WHI dataset using the WHI common ID number. Access to data from the WHI and affiliated ancillary studies can be requested by outside users. Users who want to conduct secondary analyses of this data must submit a proposal to the WHI Publications and Presentations Committee. This process maintains the confidentiality of personal information for study participants and appropriate use of these data.

#### Statistical plan

*Aim 1*. We will create age-standardized muscle mass percentile curves to characterize the distribution of D_3_Cr muscle mass in cancer survivors and non-cancer controls. We hypothesize that cancer survivors will have lower muscle mass compared to age-matched cancer-free controls. We will also develop percentile curves for women from different racial/ethnic minority groups to examine potential differences in the distribution of muscle mass among Non-Hispanic White, Non-Hispanic Black, and Hispanic women. Additionally, to examine the effect of cancer-related variables on age-standardized muscle mass among cancer survivors, we will create percentile curves stratified by time since diagnosis and treatment modality (chemotherapy yes/no). These stratified analyses will specifically address questions regarding the effect of heterogeneity of cancer type and stage of cancer at diagnosis. According to guidance from the World Health Organization and U.S. Centers for Disease control, precision of estimates for growth curves depend on the number of participants at given percentile value [35–37]. The World Health Organization suggests adequate precision for growth curve modeling is achieved with a minimum of sample size of 200 participants per percentile [35]. Based on the planned recruitment of WHI participants, we will have adequate power for this analysis.

*Aim 2*. To examine the association between D_3_Cr muscle mass and functional outcomes in cancer survivors and non-cancer controls, we will use data on physical performance (SPPB) and muscular strength (grip strength) from the WHI home visits. We hypothesize that cancer survivors will have worse physical function than non-cancer controls and will also experience greater decline in physical function over time. We will first investigate the cross-sectional joint distribution of D_3_Cr muscle mass, muscle strength, and physical function for cancer survivors and non-cancer controls. Muscle strength will be measured using handheld dynamometer (Jamar hand dynamometer; Lafayette Instruments, Lafayette, IN). Participants are instructed to squeeze the handle of the dynamometer as hard as possible for two trials per hand. Physical function is being measured via the Short Physical Performance Battery (SPPB), a validated test of physical function consisting of three tests: balance, gait speed, and chair stands. Short physical performance battery scores range from 0 to 12, with higher scores indicating better physical function. Multivariate adjusted linear regression models will be used to calculate means and 95% confidence intervals of muscle mass by SPPB score (0–12), timed walk (meters/second), number of chair stands, and grip strength (kg). These models will include a product interaction term (muscle mass x cancer survivorship status) to separately estimate these relationships among cancer survivors and non-cancer controls [38–40]. Among cancer survivors, we will examine effect heterogeneity by time since diagnosis, cancer site, and treatment modality by including product interaction terms in our fully adjusted regression models (i.e., to control for potential confounding by health or lifestyle related characteristics).

We will use annual WHI questionnaires measuring activities of daily living as the outcome in a longitudinal analysis of the relationship between baseline muscle mass and functional decline over time. Activities of daily living and instrumental activities of daily living will be measured using a validated questionnaire (RAND-36). In this analysis, we will use linear mixed effects models to examine the relationship of interest while accounting for correlation between outcomes measured on the same individual [41–43]. The model will include: year, where the coefficient represents the change in functional status per year (slope); cancer survivorship status (1 if cancer survivor, 0 if non-cancer control), where the coefficient represents the difference in mean functional status at baseline after accounting for baseline muscle mass; and a product interaction term between year and survivorship status. The interaction term can be interpreted as the difference in outcome time trend (i.e., slope) between cancer cases and non-cancer controls and will enable assessment of effect modification of the change in functional status by cancer survivorship status after accounting for baseline muscle mass. Models will be adjusted for relevant demographic and cancer-related confounding variables. Moreover, we will examine potential interaction muscle mass (at baseline) and year, to understand whether there is a difference in the slope of functional decline for each additional unit of muscle mass. This will elucidate whether there is evidence of effect modification of the change in functional decline over time by baseline muscle mass. In this analysis, we will assume linear effects but will empirically examine this assumption and employ appropriate statistical tools if evidence of non-linearity is found.

*Aim 3*. We will draw on approximately 30 years of data from WHI study participants (~1993–2023) to examine the antecedents of low muscle mass in postmenopausal women. Our primary interest in this analysis is to predict risk of having low muscle mass, defined as <10^th^ percentile muscle mass for age. We will also conduct sensitivity analyses to examine risk of being <5^th^ percentile (very low muscle mass) and <25^th^ percentile (at risk for low muscle mass). Separate risk prediction algorithms will be created for cancer survivors and non-cancer controls, and we will stratify by race/ethnicity when creating the prediction equations. We will first use multivariable logistic regression to predict the risk of low muscle mass. After standardizing predictors, the regression parameters are easily interpreted as log-odds ratios and variable importance can be assessed by comparing the magnitude of the effects. To achieve a more parsimonious model, we will use least absolute shrinkage and selection operator (LASSO) logistic regression for variable selection [44]. This approach is one in which estimation of regression coefficients is achieved by maximizing the log-likelihood of the logistic regression model that includes all variables, subject to the condition that the sum of the absolute value of the effects is smaller than a specified value, referred to as a tuning parameter [44]. As a result, the regression coefficients are continuously shrunk so that important effects stay in the model while less desirable coefficients are shrunk to zero [44]. This continuous shrinkage process results in more stable predictions than subset selection methods such as backward selection.

We will then examine whether using machine learning algorithms will improve predictive performance compared to standard approaches. The advantage of these approaches over logistic regression is that they allow for non-linear relationships between predictors and outcome and data-driven interactions that do not require a prior specification and may substantially improve predictive ability [45, 46]. Many approaches are available to analysts in standard statistical software. We propose to consider random forests [47], gradient boosting [48], and super learner [49]. Random forests and gradient boosting are ensemble methods based on building a large number of decision trees [47, 48]. In the random forest algorithm, many trees are built from resamples of the data with a random subset of the original predictors and are averaged to produce a single prediction model. In gradient boosting, a small tree is adaptively improved using resamples of the data. The super learner is an ensemble learning framework that combines predictions obtained from a range of algorithms/methods, each of which may be used to construct a prediction tool, to form a single overarching prediction tool [49]. Through theoretical work and simulations, the super learner framework has been shown to have optimal statistical properties, most notably that the final prediction tool outperforms or does no worse than any of the component algorithm/methods [49]. We will train the model on 80% of the data and validate on the remaining 20%, which is independent from the training data.

## Discussion

The remote collection of D_3_Cr muscle mass has the potential to transform our understanding of the antecedents and consequences of low muscle mass in older adults. The D_3_Cr method is a simple, inexpensive, and non-invasive assessment of muscle mass that can be used to measure muscle mass at home. This approach maximizes scientific rigor while minimizing participant burden. To our knowledge, this will be the first large-scale epidemiologic study to measure total body skeletal muscle mass remotely in older women using D_3_Cr. Ms. LILAC is well-positioned to address key unanswered questions about muscle mass in older and long-term postmenopausal cancer survivors. The WHI is the ideal setting for this study because the nested sub-cohorts (LILAC and LLS2) allow us to estimate the extent to which outcomes (i.e., decline in physical function) are specifically related to cancer or attributable to other factors. In addition, all participants recently completed a home visit funded by the WHI contract. Establishing a valid non-cancer comparison group is often a challenge in the context of cancer research; in Ms. LILAC, both cancer survivors and non-cancer controls are drawn from the same source population (WHI). This will create comparability across groups.

Avenues for future work with D_3_Cr could include its use as either an exposure (i.e., examining the relationship of D3Cr with a particular health outcome) or outcome (i.e., examining the effect of an intervention or treatment on change in D3Cr muscle mass). In particular, broadening the use of D_3_Cr has significant potential to inform the development of targeted intervention programs for older adults. As one example, D_3_Cr could be used as an outcome variable in a randomized clinical trials of a physical activity and/or dietary intervention. Successful interventions to increase muscle mass in older adults could improve quality of life, decreasing acute and chronic disease outcomes, and promoting more effective resource utilization. Future work could also examine the use of D_3_Cr in screening intervention programs in routine clinical practice (e.g., during annual physical examinations).

This protocol builds upon our successful pilot work using the remote D_3_Cr measurement protocol [22, 50]. In the pilot study, we enrolled 74 community dwelling WHI women from Buffalo, New York. We did a simple random sample of WHI participants living within 50 miles of the WHI-Buffalo clinic site. Of the 74 participants, 99% (n = 73) completed the D_3_Cr protocol; one participant had a family emergency and was not able to return her urine sample. Of note, the participant who was not able to complete the urine sample did contact the study team to inquire if she would be able to participate in the study at a later date, but due to the 4-week pilot study timeline it was not possible to have her participate. Reasons for not consenting to participate in the study included: 22% who did not consent for health-related reasons (e.g., health too poor to attend in-clinic visit, caregiving responsibilities), 12% due to scheduling, 11% due to travel or transportation concerns such as not being able to drive. (11% does not drive, lives too far). Only 3% (n = 5) reported not participating because they did not want to take the D_3_Cr pill. In the pilot study, all samples received from the study participants were received in good condition, demonstrating the effectiveness of our shipping protocol. This pilot work demonstrates feasibility of the remote D_3_Cr-protocol as well as acceptability (all study participants agreed to take the D_3_Cr pill provided); safety (no participant had difficulty swallowing the D_3_Cr pill or reported any side effects); high-return rate; and very good sample viability. Analyses of pilot data demonstrated a clear relationship between muscle mass, measured by D_3_Cr, and physical function [22]. These findings were broadly consistent with measures of D_3_Cr and physical performance in older men reported by Cawthon and colleagues [20].

As with any study protocol involving primary data collection, potential problems may arise. Investigators using the D_3_Cr protocol must carefully consider alternative strategies should they be necessary. Participant non-response and loss-to-follow-up are concerns in any prospective study design, especially in older adults. The WHI has a long history of exceptional follow-up and participant retention, including a very small proportion of loss-to-follow-up. Despite the success of sample return in our pilot study, we anticipate some participants will not collect the urine sample or send it back during the required time frame. Also, in our experience, it can be confusing to schedule a pickup by telephone because automatic voice-prompt software may be challenging to navigate. We will provide participants with a pre-addressed return mailing label and extremely detailed, step-by-step written instructions as we did successfully in the pilot study. We will use a large type font (size 14) for all instructions and mailings in case there are participants with poor eyesight. If a participant is having a difficult time scheduling the sample pick up, we will instruct them to call the study team and we will schedule the pick-up for them. Importantly, in our pilot study, nearly all participants successfully called to schedule a pick up; only 2 women required assistance from the WHI Buffalo clinic staff to schedule the pickup.

The D_3_Cr method has the potential to create a new area of scientific inquiry and significantly advance our understanding of muscle mass in the context of aging and cancer research. The remote D_3_Cr measurement approach developed in Ms. LILAC broad applications across epidemiology, public health, and clinical medicine. Sharing the Ms. LILAC study protocol will make this approach more accessible to researchers in a wide range of contexts.

## Supporting information

S1 File Supplementary documentation describing the D_3_Cr remote collection protocol. (PDF)

## References

[1] Hubbard JM, Cohen HJ, Muss HB: Incorporating biomarkers into cancer and aging research. *Journal of Clinical Oncology* 2014, 32(24):2611–2616. doi: 10.1200/JCO.2014.55.4261 25071114 PMC4876339

[2] Caan BJ, Cespedes Feliciano EM, Prado CM, Alexeeff S, Kroenke CH, Bradshaw P, et al: Association of Muscle and Adiposity Measured by Computed Tomography With Survival in Patients With Nonmetastatic Breast Cancer. *JAMA oncology* 2018, 4(6):798–804. doi: 10.1001/jamaoncol.2018.0137 29621380 PMC6584322

[3] Rier HN, Jager A, Sleijfer S, Maier AB, Levin MD: The Prevalence and Prognostic Value of Low Muscle Mass in Cancer Patients: A Review of the Literature. *The oncologist* 2016. doi: 10.1634/theoncologist.2016-0066 27412391 PMC5189631

[4] Shachar SS, Williams GR, Muss HB, Nishijima TF: Prognostic value of sarcopenia in adults with solid tumours: A meta-analysis and systematic review. *EurJCancer* 2016, 57:58–67. doi: 10.1016/j.ejca.2015.12.030 26882087

[5] Caan BJ, Meyerhardt JA, Kroenke CH, Alexeeff S, Xiao J, Weltzien E, et al: Explaining the Obesity Paradox: The Association between Body Composition and Colorectal Cancer Survival (C-SCANS Study). *Cancer Epidemiology Biomarkers & Prevention* 2017, 26(7):1008–1015. doi: 10.1158/1055-9965.EPI-17-0200 28506965 PMC5647152

[6] Hoppe S, Rainfray M, Fonck M, Hoppenreys L, Blanc JF, Ceccaldi J, et al: Functional decline in older patients with cancer receiving first-line chemotherapy. *J Clin Oncol* 2013, 31(31):3877–3882. doi: 10.1200/JCO.2012.47.7430 24062399

[7] Cawthon PM, Visser M, Arai H, Ávila-Funes JA, Barazzoni R, Bhasin S, et al: Defining terms commonly used in sarcopenia research: a glossary proposed by the Global Leadership in Sarcopenia (GLIS) Steering Committee. *Eur Geriatr Med* 2022, 13(6):1239–1244. doi: 10.1007/s41999-022-00706-5 36445639 PMC9722886

[8] Cespedes Feliciano EM, Lee VS, Prado CM, Meyerhardt JA, Alexeeff S, Kroenke CH, et al: Muscle mass at the time of diagnosis of nonmetastatic colon cancer and early discontinuation of chemotherapy, delays, and dose reductions on adjuvant FOLFOX: The C-SCANS study. *Cancer* 2017, 123(24):4868–4877. doi: 10.1002/cncr.30950 28881381 PMC5716836

[9] Looijaard SMLM, te Lintel Hekkert ML, Wüst RCI, Otten RHJ, Meskers CGM, Maier AB: Pathophysiological mechanisms explaining poor clinical outcome of older cancer patients with low skeletal muscle mass. *Acta Physiologica* 2021, 231(1):e13516. 32478975 10.1111/apha.13516 PMC7757176

[10] Pratesi A, Tarantini F, Di Bari M: Skeletal muscle: an endocrine organ. *Clin Cases Miner Bone Metab* 2013, 10(1):11–14. doi: 10.11138/ccmbm/2013.10.1.011 23858303 PMC3710002

[11] Vega MC, Laviano A, Pimentel GD: Sarcopenia and chemotherapy-mediated toxicity. *Einstein (Sao Paulo*, *Brazil)* 2016, 14(4):580–584. doi: 10.1590/S1679-45082016MD3740 28076611 PMC5221390

[12] Prado CM, Cushen SJ, Orsso CE, Ryan AM: Sarcopenia and cachexia in the era of obesity: clinical and nutritional impact. *The Proceedings of the Nutrition Society* 2016, 75(2):188–198. doi: 10.1017/S0029665115004279 26743210

[13] Rowland JH, Bellizzi KM: Cancer survivorship issues: life after treatment and implications for an aging population. *J Clin Oncol* 2014, 32(24):2662–2668. doi: 10.1200/JCO.2014.55.8361 25071099 PMC4164810

[14] Buckinx F, Aubertin-Leheudre M: Sarcopenia in Menopausal Women: Current Perspectives. *Int J Womens Health* 2022, 14:805–819. doi: 10.2147/IJWH.S340537 35769543 PMC9235827

[15] McCarthy C, Schoeller D, Brown JC, Gonzalez MC, Varanoske AN, Cataldi D, et al: D3-creatine dilution for skeletal muscle mass measurement: historical development and current status. *Journal of Cachexia*, *Sarcopenia and Muscle* 2022, 13(6):2595–2607. 10.1002/jcsm.13083 PMC9745476 36059250

[16] Heymsfield SB, Adamek M, Gonzalez MC, Jia G, Thomas DM: Assessing skeletal muscle mass: historical overview and state of the art. *Journal of Cachexia*, *Sarcopenia and Muscle* 2014, 5(1):9–18. doi: 10.1007/s13539-014-0130-5 24532493 PMC3953319

[17] Evans WJ, Hellerstein M, Orwoll E, Cummings S, Cawthon PM: D3-Creatine dilution and the importance of accuracy in the assessment of skeletal muscle mass. *Journal of Cachexia*, *Sarcopenia and Muscle* 2019, 10(1):14–21. 10.1002/jcsm.12390 PMC6438329 30900400

[18] Shah UA, Ballinger TJ, Bhandari R, Dieli-Conwright CM, Guertin KA, Hibler EA, et al: Imaging modalities for measuring body composition in patients with cancer: opportunities and challenges. *JNCI Monographs* 2023, 2023(61):56–67. 10.1093/jncimonographs/lgad001 PMC10157788 37139984

[19] Cawthon PM, Orwoll ES, Peters KE, Ensrud KE, Cauley JA, Kado DM, et al: Strong Relation between Muscle Mass Determined by D3-creatine Dilution, Physical Performance and Incidence of Falls and Mobility Limitations in a Prospective Cohort of Older Men. *Journals of Gerontology*: *Series A* 2019, 74(6):844–852. 10.1093/gerona/gly129 PMC6521914 29897420

[20] Cawthon PM, Blackwell T, Cummings SR, Orwoll ES, Duchowny KA, Kado DM, et al: Muscle mass assessed by D3-Creatine dilution method and incident self-reported disability and mortality in a prospective observational study of community dwelling older men. *The Journals of Gerontology*: *Series A* 2020. 10.1093/gerona/glaa111 PMC7756711 32442245

[21] Clark RV, Walker AC, O’Connor-Semmes RL, Leonard MS, Miller RR, Stimpson SA, et al: Total body skeletal muscle mass: estimation by creatine (methyl-d3) dilution in humans. *Journal of Applied Physiol (1985)* 2014, 116(12):1605–1613. doi: 10.1152/japplphysiol.00045.2014 24764133 PMC4064374

[22] Zhu K, Wactawski-Wende J, Ochs-Balcom HM, LaMonte MJ, Hovey KM, Evans W, et al: The Association of Muscle Mass Measured by D3-Creatine Dilution Method With Dual-Energy X-Ray Absorptiometry and Physical Function in Postmenopausal Women. *J Gerontol A Biol Sci Med Sci* 2021, 76(9):1591–1599. doi: 10.1093/gerona/glab020 33475725 PMC8361359

[23] Stimpson SA, Turner SM, Clifton LG, Poole JC, Mohammed HA, Shearer TW, et al: Total-body creatine pool size and skeletal muscle mass determination by creatine-(methyl-d3) dilution in rats. *Journal of Applied Physiology* 2012, 112(11):1940–1948. doi: 10.1152/japplphysiol.00122.2012 22422801

[24] Shankaran M, Czerwieniec G, Fessler C, Wong PyA, Killion S, Turner SM, et al: Dilution of oral D(3)‐Creatine to measure creatine pool size and estimate skeletal muscle mass: development of a correction algorithm. *JCachexia*, *Sarcopenia and Muscle* 2018, 9(3):540–546. 10.1002/jcsm.12278 PMC5989770 29663711

[25] Guida JL, Ahles TA, Belsky D, Campisi J, Cohen HJ, DeGregori J, et al: Measuring Aging and Identifying Aging Phenotypes in Cancer Survivors. *JNCI*: *Journal of the National Cancer Institute* 2019, 111(12):1245–1254. doi: 10.1093/jnci/djz136 31321426 PMC7962788

[26] Hays J, Hunt JR, Hubbell FA, Anderson GL, Limacher M, Allen C, et al: The Women’s Health Initiative recruitment methods and results. *Ann Epidemiol* 2003, 13(9 Suppl):S18–77. doi: 10.1016/s1047-2797(03)00042-5 14575939

[27] The Women’s Health Initiative Study G: Design of the Women’s Health Initiative Clinical Trial and Observational Study. *Controlled Clinical Trials* 1998, 19(1):61–109. 9492970 10.1016/s0197-2456(97)00078-0

[28] Paskett ED, Caan BJ, Johnson L, Bernardo BM, Young GS, Pennell ML, et al: The Women’s Health Initiative (WHI) Life and Longevity After Cancer (LILAC) Study: Description and Baseline Characteristics of Participants. *Cancer Epidemiology Biomarkers &Prevention* 2018, 27(2):125–137. doi: 10.1158/1055-9965.EPI-17-0581 29378785 PMC5809310

[29] LaCroix AZ: THE WOMEN’S HEALTH INITIATIVE (WHI): STILL LEARNING FROM 161,808 POSTMENOPAUSAL WOMEN. *Innovation in Aging* 2019, 3(Supplement_1):S355–S355.

[30] Guralnik JM, Ferrucci L, Pieper CF, Leveille SG, Markides KS, Ostir GV, et al: Lower Extremity Function and Subsequent Disability: Consistency Across Studies, Predictive Models, and Value of Gait Speed Alone Compared With the Short Physical Performance Battery. *The Journals of Gerontology*: *Series A* 2000, 55(4):M221–M231. doi: 10.1093/gerona/55.4.m221 10811152 PMC12149745

[31] Woods NF, Rillamas-Sun E, Cochrane BB, La Croix AZ, Seeman TE, Tindle HA, et al: Aging Well: Observations From the Women’s Health Initiative Study. *The journals of gerontology Series A*, *Biological sciences and medical sciences* 2016, 71 Suppl 1:S3–S12. 26858322 10.1093/gerona/glv054 PMC5865531

[32] Cormier JN, Davidson L, Xing Y, Webster K, Cella D: Measuring quality of life in patients with melanoma: development of the FACT-melanoma subscale. *J Support Oncol* 2005, 3(2):139–145. 15796446

[33] Lloyd-Jones DM, Hong Y, Labarthe D, Mozaffarian D, Appel LJ, Van Horn L, et al: Defining and setting national goals for cardiovascular health promotion and disease reduction: the American Heart Association’s strategic Impact Goal through 2020 and beyond. *Circulation* 2010, 121(4):586–613. doi: 10.1161/CIRCULATIONAHA.109.192703 20089546

[34] Yost KJ, Cheville AL, Weaver AL, Al Hilli M, Dowdy SC: Development and validation of a self-report lower-extremity lymphedema screening questionnaire in women. *Phys Ther* 2013, 93(5):694–703. doi: 10.2522/ptj.20120088 23288911

[35] Guo SS, Roche AF, Chumlea WC, Johnson C, Kuczmarski RJ, Curtin R: Statistical effects of varying sample sizes on the precision of percentile estimates. *Am J Hum Biol* 2000, 12(1):64–74. doi: 10.1002/(SICI)1520-6300(200001/02)12:1&lt;64::AID-AJHB8&gt;3.0.CO;2-N 11534005

[36] Kuczmarski RJ, Ogden CL, Guo SS, Grummer-Strawn LM, Flegal KM, Mei Z, et al: 2000 CDC Growth Charts for the United States: methods and development. *Vital and health statistics Series 11*, *Data from the national health survey* 2002(246):1–190. 12043359

[37] GROUP WMGRS, de Onis M: WHO Child Growth Standards based on length/height, weight and age. *Acta Paediatrica* 2006, 95(S450):76–85. doi: 10.1111/j.1651-2227.2006.tb02378.x 16817681

[38] Greenland S: Interactions in epidemiology: relevance, identification, and estimation. *Epidemiology* 2009, 20(1):14–17. doi: 10.1097/EDE.0b013e318193e7b5 19234397

[39] Greenland S, Rothman KJ: Concepts of Interaction. In: *Modern Epidemiology*. edn. Edited by Rothman KJ, Greenland S. Philadelphia, Pa.: Lippincott-Raven; 1998.

[40] Knol MJ, van der Tweel I, Grobbee DE, Numans ME, Geerlings MI: Estimating interaction on an additive scale between continuous determinants in a logistic regression model. *International journal of epidemiology* 2007, 36(5):1111–1118. doi: 10.1093/ije/dym157 17726040

[41] Goldstein H, Carpenter J, Kenward MG, Levin KA: Multilevel models with multivariate mixed response types. *Statistical Modelling* 2009, 9(3):173–197.

[42] Lai Y, Albert PS: Identifying multiple change points in a linear mixed effects model. *Statistics in medicine* 2014, 33(6):1015–1028. doi: 10.1002/sim.5996 24114935 PMC3971951

[43] Nguyen DV, Sentürk D, Carroll RJ: Covariate-Adjusted Linear Mixed Effects Model with an Application to Longitudinal Data. *J Nonparametr Stat* 2008, 20(6):459–481. doi: 10.1080/10485250802226435 19266053 PMC2650843

[44] Tibshirani R: Regression Shrinkage and Selection Via the Lasso. *Journal of the Royal Statistical Society*: *Series B (Methodological)* 1996, 58(1):267–288.

[45] Couronné R, Probst P, Boulesteix A-L: Random forest versus logistic regression: a large-scale benchmark experiment. *BMC Bioinformatics* 2018, 19(1):270. doi: 10.1186/s12859-018-2264-5 30016950 PMC6050737

[46] Natekin A, Knoll A: Gradient boosting machines, a tutorial. *Front Neurorobot* 2013, 7:21–21. doi: 10.3389/fnbot.2013.00021 24409142 PMC3885826

[47] Breiman L: Random Forests. *Machine Learning* 2001, 45(1):5–32.

[48] Friedman JH: Greedy Function Approximation: A Gradient Boosting Machine. *The Annals of Statistics* 2001, 29(5):1189–1232.

[49] van der Laan MJ, Polley EC, Hubbard AE: Super learner. *Stat Appl Genet Mol Biol* 2007, 6:Article25. doi: 10.2202/1544-6115.1309 17910531

[50] Banack HR, LaMonte MJ, Manson JE, Zhu K, Evans WJ, Shankaran M, et al: Association of muscle mass measured by D3-Creatine (D3Cr), sarcopenic obesity, and insulin-glucose homeostasis in postmenopausal women. *PLOS ONE* 2022, 17(12):e0278723. doi: 10.1371/journal.pone.0278723 36490255 PMC9733841

