# Supplementary material for: A protocol for remote collection of skeletal muscle mass via D3-creatine dilution in community-dwelling postmenopausal women from the Women’s Health Initiative
Source: PLoS One. 2024 Apr 17;19(4):e0300140. doi: 10.1371/journal.pone.0300140 (PMC11023459; doi:10.1371/journal.pone.0300140)
Supplement: S1 File — (PDF) [file pone.0300140.s001.pdf]

Schematic illustration of the protocol for the Ms. LILAC study

Participant receives study kit via mail

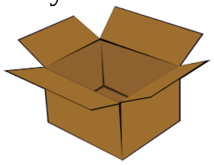

→

Participant ingests the D_3Cr capsule provided in the study kit

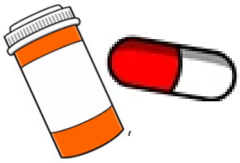

↓

Participant calls toll-free number to study team to report taking pill and receive specific instructions about when to do urine sample

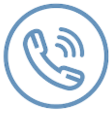

↓

Participant receives a reminder call the night before they are required to provide their urine sample

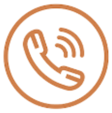

↓

Participant collects urine sample using dipstick provided in study kit 48-96 hours after ingesting the D_3Cr capsule

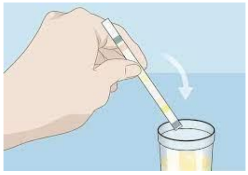

↓

Participant packs urine dipstick into return shipping container provided and calls courier to schedule same-day pick up

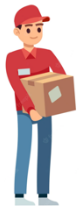

{DATE}

Mrs. (or other title)
Street
City, State ZIP

Dear ____,

We are contacting you today about a research study we are conducting on muscle mass in postmenopausal women. In this study we will be using a simple method for measuring muscle mass called the creatine dilution method.

Let us briefly describe our study to you so you can decide if you might be interested in participating. If you agree to take part, we will send you a small study kit in the mail with a pill made of creatine and other supplies required for our study. We will provide you with specific instructions for taking the pill. Two to four days later, we will ask you to provide a urine sample at home using the supplies from in the study kit. We will then ask you to send the urine sample back to us. **There are no known risks associated with taking this creatine pill.** Creatine is a normal component of a healthy diet, found primarily in meat and other protein sources. The average person consumes approximately 1 gram of creatine per day; that is many times greater than the amount of creatine you will receive for this study. In total, we estimate that your participation in this study will take approximately 20-30 minutes.

Enclosed you will find an informed consent form. If you are interested in participating in this study, please review and sign the consent form, place in the pre-paid mailing envelope, and mail it back to our study team. After we receive your consent form, we will send you the study kit. If you would like to speak to someone in the meantime to learn more about the study or if you have any questions, please call our study team directly.

We look forward to hearing from you soon!

Sincerely,

Thank you for participating in our research study. Please read all instructions thoroughly before proceeding.

As we described in our invitation letter, we have included all the supplies required to complete the study in this kit. As part of this study you will:
1) take a creatine pill,
2) provide a fasting urine sample, and
3) send the urine sample back to our clinic via FedEx.

In this kit, you will find:

▪ Creatine pill in small, labeled plastic vial (located inside urine container)
▪ Urine collection container
▪ One pair of gloves
▪ Return FedEx mailer with silver insulated protective mailer inside
▪ A zip-top plastic bag containing dipstick (inside return mailer)
▪ Cold packs (inside return mailer)
▪ Sample collection form
▪ Sample collection instructions, attached here

Specific Instructions:

- Take the creatine pill on **Saturday morning**. You do not need to fast prior to taking the pill; you can eat your breakfast as normal and take all your required medications.

- Provide your urine sample using the supplies in this kit **before 10:00 AM on Tuesday morning.** We provide specific instructions on how to do your urine sample collection in the enclosed instructions.

- **You should not eat anything for eight (8) hours before you provide your urine sample.** Please provide your sample before eating breakfast.

OVER →

You may drink water, but do not drink coffee, tea, or other liquid before providing your sample.

- We recognize that many people wake during the night or early morning to urinate. You do not need to collect your urine sample in the middle of the night. On Tuesday, we ask that you collect your sample once you wake up for the day, whatever time that may be, as long as you do so before eating or drinking.

- ****Important: Please place the cold packs (located inside the return envelope) in the freezer now so they will be frozen by the time you provide your urine sample on Tuesday.**

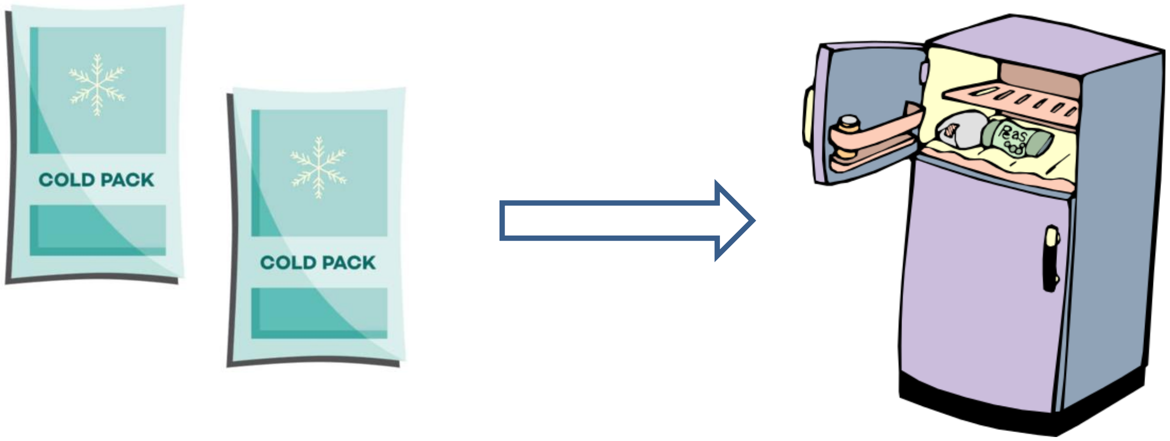

SAMPLE COLLECTION INSTRUCTIONS

When you are ready to prepare the urine sample, please do the following:

1. Have equipment ready:

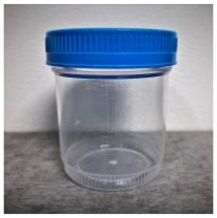

Urine collection cup

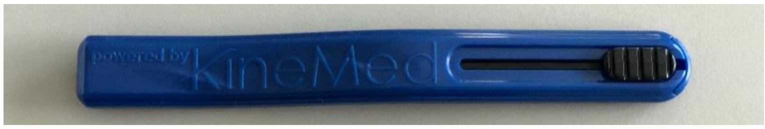

Dipstick

2. Starting midway through urination, collect urine using the collection cup provided. Set urine collection cup on counter or other flat surface. You may use the disposable gloves provided if you would like (optional).

3. Push up on tab on dipstick to reveal white filter paper.

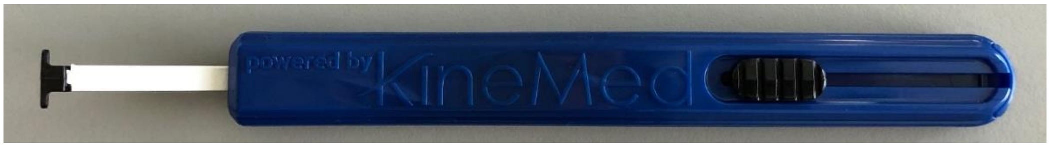

4. Dip white filter paper into urine collected in cup. **<u>Please try to submerge filter paper if possible.</u>** Then, retract filter paper back into the plastic cartridge.

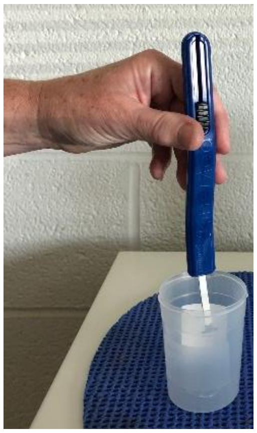

5. Carefully seal the blue dipstick in the small zip top bag. You may now dispose of the liquid urine and collection cup.

6. Complete the sample collection form.

OVER →

Sample Collection Form

Please record the date you provided your **urine sample**:

Date: ______/______/______
Month Day Year

Fill in date here

PLEASE MAKE SURE TO RETURN THIS PAPER WITH YOUR SPECIMEN

7. Place the zip top bag (including dipstick) inside the silver insulated mailing package **with the cold packs.** This is what it will look like once you have packed the dipstick and ice packs into the silver insulated mailing package:

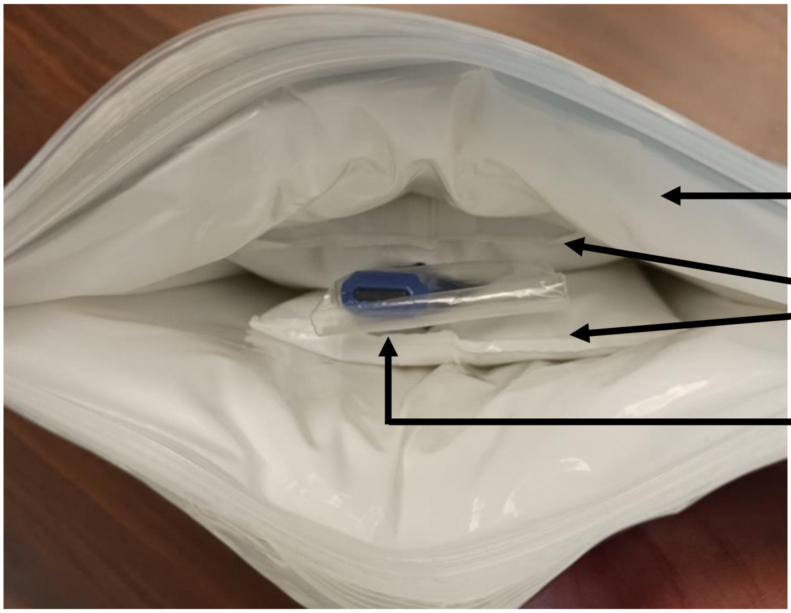

8. Place the silver package inside the FedEx mailing envelope to return to the study team. Remember to include your sample collection form. Use the adhesive strip to seal your FedEx package. Before sealing, please ensure your FedEx mailing envelope contains:

☐ Insulated package with cold packs and dipstick inside

☐ Completed sample collection form

(See next page for picture)

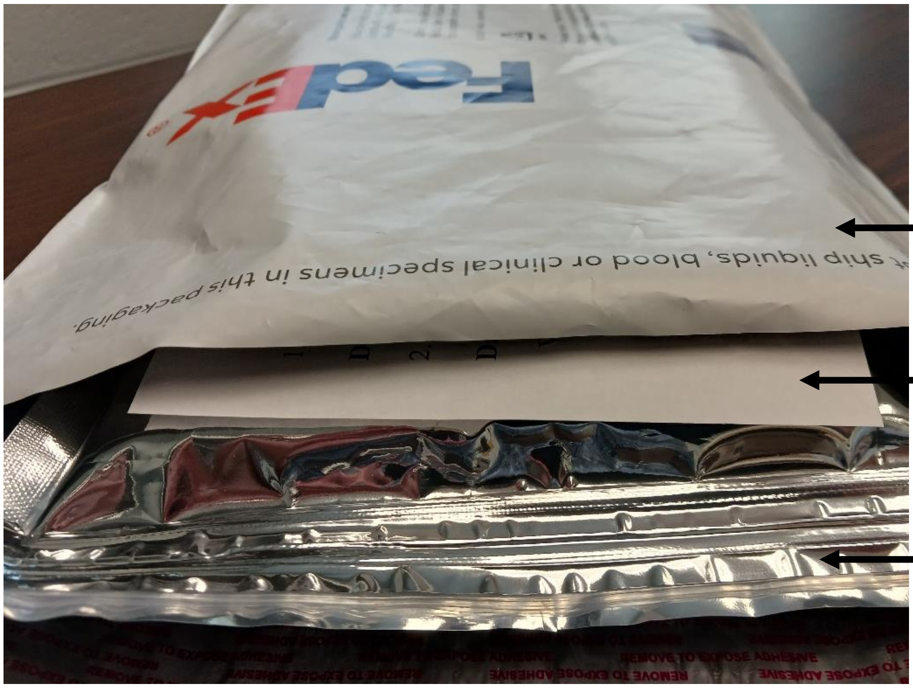

9. Call 1-800-463-3339 to schedule a pick up by FedEx.

When possible, please try to call FedEx **<u>before 12:00pm</u>** (noon) the day you provide your sample to ensure a same-day pick-up.

If you experience any difficulties scheduling the FedEx pick-up, please call our study team for assistance.

STUDY SCHEDULE

PLEASE PUT THIS ON YOUR REFRIGERATOR OR ANOTHER PROMINENT PLACE

SATURDAY

MORNING:

TAKE CREATINE PILL FROM STUDY KIT

SUNDAY

MONDAY

AFTERNOON:

EXPECT PHONE CALL FROM STUDY TEAM

FAST OVERNIGHT (PRIOR TO URINE COLLECTION)

TUESDAY

MORNING:

- COLLECT URINE SAMPLE
- SEND SAMPLE VIA FEDEX

WEDNESDAY

THURSDAY

FRIDAY

REMINDER!

PLEASE DO NOT EAT OR DRINK ANYTHING BEFORE TAKING YOUR URINE SAMPLE!
